# Lapachol-Induced Upregulation of Sirt1/Sirt3 is linked with Improved Skin Wound Healing in Alloxan-induced Diabetic Mice

**DOI:** 10.22037/ijpr.2021.112722.13914

**Published:** 2021

**Authors:** Shaheen Bibi, Fayyaz Ahmad, Muhammad Rizwan Alam, Muhammad Ansar, Kim Sun Yeou, Hussain Mustatab Wahedi

**Affiliations:** a *Department of Biochemistry, Faculty of Biological Sciences, Quaid-i-Azam, University, Islamabad, 45320, Pakistan. *; b *College of Pharmacy, Gachon University, #191, Hambakmoero, Yeonsu-gu, Incheon 21936, Republic of Korea. *; c *Department of Biological Sciences, National University of Medical Sciences, C/O Military Hospital, The Mall Road, 46000 Rawalpindi, Pakistan.*

**Keywords:** Lapachol, Sirt1, Sirt3, Diabetic wound healing, Skin

## Abstract

Timely repair of damaged skin is very important to maintain the integrity and homeostasis of skin, but the wound healing process is compromised in diabetic patients due to several extrinsic and intrinsic factors thus lead to leg amputation and death eventually. Sirtuins, a family of seven conserved proteins are known to be associated with pathophysiological processes of the skin. The most important among them are sirt1and sirt3 involved in cell regeneration and cell survival. Naphthoquinone derivatives have a wide range of therapeutic properties, but the potential diabetic wound healing activity of lapachol has not been identified yet. The present study thus aimed to investigate the wound healing effects of lapachol in a diabetic mouse model. Diabetic wounded mice were divided into 3 groups; vehicle, lapachol 0.05%, and lapachol 0.1%. Skin samples collected from diabetic wounded mice on different time points after treatment for 10 consecutive days were subjected to downstream analysis by western blot, ELISA and histology. Lapachol treatment was found to enhance the expression of sirt1/sirt3 and other proteins involved in cell migration and blood vessel formation. The tissue development rate was increased by lapachol treatment with better collagen deposition. Interestingly, lapachol treatment also gave rise to a high concentration of growth factors resulting in speedy and timely recovery of injured skin. In summary, our findings suggest that lapachol promotes efficient wound healing in a diabetic mouse model by increasing the expression of sirt1 and sirt3 and other proteins related to wound repair and skin regeneration including α-PAK, RAC1/CDC42, VEGF and growth factors viz PDGF and VEGF. This research work finds a novel potential activator of sirtuins in the form of lapachol and depicts the role of activated sirtuins in diabetic wound healing.

## Introduction

The process of wound healing is very complex having many overlapping steps that involve blood clotting, inflammation, re-epithelialization, and tissue remodeling (1, 2). There are several chemokines, growth factors, and cytokines involved in this complex process of wound closure (2). Blood clotting starts immediately after wound creation (3). The platelets trapped inside the blood clot play a very important role in wound healing, as their cytoplasm is enriched with growth factors containing alpha granules. Some of the growth factors like PDGF, EGF, and TGF-β provide the basis for the recruitment of various cells required for efficient wound healing (3, 4). The inflammatory phase provides an immunological barrier against attacking pathogens (5). The neutrophils infiltrated to the wound site during the inflammatory phase are very important for the removal of bacteria and other invading microorganisms through the process of phagocytosis (6). The late inflammatory response is marked by the recruitment of macrophages which act as a potent reservoir of tissue growth factors (7). Once the blood clotting has taken place and pathogens have been removed from the injury site, tissue repair begins (8). Keratinocytes are first polarized, then migrated during the proliferative phase, followed by the proliferation of endothelial cells and angiogenesis (9). In the last step, *i.e. *remodeling, the quantity of growth factor and cytokine-producing cells (macrophages and neutrophils) and fibroblasts starts to decrease; though, fibroblasts last to produce collagen, which is the most important step of the remodeling phase (2).

A report published by World Health Organization shows that every year, about one million people loss their life due to diabetes (10). The pathophysiologic link between diabetes and delayed wound healing is very complicated (11). There are hundreds of physiological factors that are known to contribute to defective wound healing. One of the major factors is less or impaired production of growth factors (12, 13). Other factors are decreased angiogenic response, poor granulation tissue formation, defective function of the epidermal barrier, accumulation, and impaired function of collagen accompanied by poor migration of fibroblasts and keratinocytes (14-16).

Sirtuins belong to a family of enzymes that fall in class III histone deacetylases (17). The most important events of wound healing are inflammation, cell division, and cell migration. Sirtuins enhance wound healing by promoting cell proliferation and cell migration along with their anti-inflammatory effects (18).

Sirt1 is involved in mediating proangiogenic signals in endothelial cells thus increasing the capillary density and blood flow (19). Sirt1 is involved in regulating cell migration and promoting epithelial regeneration (20, 21). Epidermal sirt1 is important for efficient wound healing (22). It is reported previously that increasing sirt1 expression in normal and ultraviolet B (UV-B) irradiated conditions by juglone resulted in skin cells regeneration and repair (23). Sirt3 also belongs to the sirtuin family of enzymes. It is present in mitochondria and performs de-acetylation of proteins localized in mitochondria (24). Sirt3 maintains mitochondrial integrity and prevents cell death induced by stress (25, 26). 

The natural and synthetic derivatives of naphthoquinone can interfere with the biological activities of topoisomerases, enzymes that are involved in DNA replication. Due to these properties, the quinone derivatives are used as drugs against many tropical diseases. A yellow-colored matter known as lapachol is present in grains of many wooden trees. It is a prenyl naphthoquinone and is extracted from plants belonging to the *Bignoniaceae* family (27). There are numbers of therapeutic activities attributed to lapachol and its derivatives such as antitumor, antiviral, antifungal, antiseptic, antibacterial, antimalarial and anti-inflammatory (28). These naturally growing naphthoquinones have been found to possess wound healing activities, which is most probably because of their anti-oxidative and anti-inflammatory properties (29, 30). Another study has reported the diabetic wound healing properties of naphthoquinone derivatives but they did not investigate the diabetic wound healing property of lapachol (31). For this reason, we wanted to check the wound healing properties of lapachol in diabetic mice, because it has not studied before. 

About 15% of the total of 200 million diabetic patients is suffering from diabetic foot ulcer around the globe (32). There is no such promising cure for diabetic wound healing. The newly established drugs are either very costly or have some side effects (33). Thus, it is time to come up with medicines which are cost-effective as well as without harmful effects. Considering these issues, we designed the present study to find out the wound healing effects of lapachol in diabetic mice and to find a novel sirtuin activator. Thus we checked the effect of different concentrations of lapachol on the expression of sirt1 and sirt3. In addition, we also wanted to examine its impact on other wound-healing-related proteins including α-PAK, VEGF, PDGF, EGF and CDC42/Rac1.

## Experimental

*Mice Grouping and Diabetes Induction*

All the procedures with animals were performed in adherence to the Principles of Laboratory Animal Care (NIH publication #85−23, revised in 1985). The compound lapachol was commercially purchased from Sigma Aldrich. Swiss albino mice 6-7 weeks old and 30-40 g in weight (Purchased from National Institute of Health, Islamabad) were divided randomly into 3 groups: vehicle, lapachol 0.05%, and lapachol 0.1% having 5 mice per group and were kept in a humidity and a temperature-controlled room with 12 h light and dark cycles with free access to food and water. After one week of acclimatization, basal blood glucose of mice from each group was randomly checked by using a one-touch blood glucometer (Abbot) and was found to be around 120-140 mg/dL. To induce diabetes, mice were starved overnight, followed by one-time intraperitoneal alloxan injection prepared in normal saline (200 mg/kg). 20% sucrose solution was available in each cage to prevent the death of mice by hypoglycemic shock. Food was restored and sucrose water was replaced with normal water after 12 h. Mice showing a blood glucose level of 300 mg/dL a few days’ post-injection was considered diabetic. 

*Wound Creation and Drug Treatment*

The dorsal posterior region of each mouse was made hairless by using hair-removing cream. Mice were anesthetized by chloroform and two full-thickness wounds of equal size were created by a 4mm biopsy punch on the sterilized dorsal posterior region of the mouse. Mice were topically treated with 200 μL of 0.1% and 0.05% solutions of lapachol prepared in a mixture of propane diol, ethanol, and distilled water (5:3:2 respectively) per mouse once per day for 10 days post-wounding. Mice from the vehicle group were treated with solvent only. The wounded area of mice was photographed with a digital camera from day 0 to day 10 post-wounding. 

*Histological Assessment*

Five mice from each group were sacrificed on days 3, 7 and 10 post-injuries. Skin samples containing the wounded area were collected immediately and preserved in 10% formalin, followed by embedding in paraffin wax and cutting into 5µm thick sections to check the epidermis regeneration by H&E staining and collagen deposition by trichrome staining.

*Western Blotting*

To check the expression of sirt1 and sirt3 along with different other proteins involved in wound healing, western blotting was performed by using skin tissue lysates. Skin samples from five mice per group were collected on days 3^rd^, 7^th^ and 10^th ^and were proceeded for a western blot on the very next day of collection. Proteins were extracted from skin tissue using RIPA buffer. The lysis buffer contained: 10 mM Tris-Hcl (pH 8), 1 mM EDTA, 140 mM NaCl ,1% Triton X-100, 0.1% SDS, 1% protease inhibitor, 1% phosphatase inhibitor, 0.01% sodium deoxycholate. The extracted protein was separated on SDS-PAGE followed by transfer of separated proteins on nitrocellulose membrane (GE Healthcare, Life Sciences) and incubated with desired primary antibodies diluted 1:1000 times overnight at 4 °C. The primary antibodies used were VEGF (Santa Cruz U.S.A), SIRT 1 (Santa Cruz U.S.A), SIRT 3 (Santa Cruz U.S.A), α-Pak (Santa Cruz U.S.A), Cdc42 (Santa Cruz U.S.A), and Rac1/2/3 (E-AB. The complex of primary antibody-antigen was confirmed by treating with horseradish peroxidase-conjugated secondary antibodies (1:5000 dilution fold) including mouse anti-rabbit IgG (Santa Cruz U.S.A) and m-IgGk (Santa Cruz U.S.A) for 1 hour. Bands were visualized by a gel documentation system (Alpha View SA Version 3.4.0.0). α-Tubulin (Santa Cruz U.S.A) was used as a loading control.

*Enzyme-linked immunosorbent assay*

The concentration of growth factors EGF and PDGF were measured by using ELISA kits (Mouse EGF) and (Mouse PDGF) respectively according to the manufacturer’s instructions. The proteins were extracted on the very next day after sacrificing 5 mice from each group and skin sample collection. Briefly, the plate (Thermo Fisher SCIENTIFIC) was incubated with capture antibody (R&D Systems, Inc) overnight followed by three times wash with washing buffer (0.05% Tween-20 dissolved in PBS). Blocking of the plate was carried out by reagent diluent (1% BSA in PBS), aspirated and then the plate was coated with standards (R&D Systems, Inc) and protein sample for 2 h. Detection antibody (844336 R&D Systems, Inc) was used to form a complex with the desired protein and the complex was then treated with streptavidin-HRP (R&D Systems, Inc) followed by stop solution (R&D Systems, Inc). The optical density was determined at 450 nm by using Multiscan Go (Thermo Scientific, Japan).

*Statistical Analysis*

To find the significant difference among the groups, various statistical tests such as student T-test and ANOVA along with multiple comparison tests including Tukey’sposthoc test were applied using GraphPad Prism5 software. All the data were presented as mean ± SD. *P-*values were taken statistically significant when less than 0.05.

## Results

*Lapachol Enhances Wound Healing and Sirt1/Sirt3 Expression in Mice*

**Figure 1:**
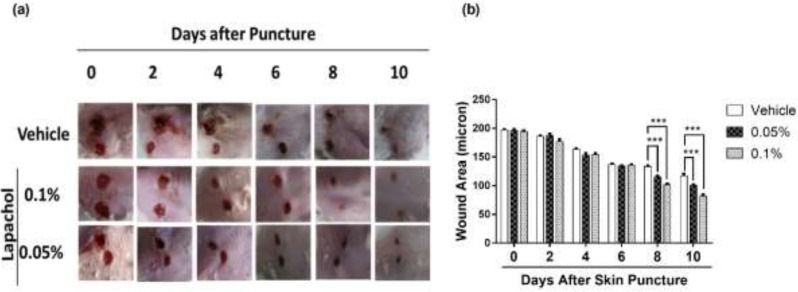
Effect of lapachol on diabetic wound healing and sirt1/sirt3 expression. Five mice from each group were photographed digitally and the skin sample was collected from each group (a) Representative images of wounded skin from each group over 10 days’ period post-wounding. (b) Graphical representation of average wound area in different groups on different days’ post-injury.

By looking at the photographs from 10 consecutive days, we found that the rate of wound closure was significantly higher in mice treated with lapachol as compared to the mice treated with vehicle only (Figure 1A). This difference in the rate of wound closure was prominent from day 6 onwards. There was a significant reduction of wound size in lapachol treated mice on days 8 and 10 of treatment compared to the vehicle (Figure 1A). The difference between the wound size of lapachol treated mice in comparison to a vehicle can be seen clearly in the figure. Moreover, the difference was analyzed statistically and it proved to be significantly higher than the vehicle-treated group (Figure 1B). Mice in the 0.1% lapachol treated group showed the fastest wound healing with complete wound closure on day 10^th^ of treatment. We found that mice treated with 0.05% and 0.1% lapachol had perfectly healed wounds compared to the mice group treated with the solvent only. Herein, we got the first clue of lapachol being responsible somehow for the wound healing in diabetic mice, because the vehicle group didn’t show the promising result. We carried on our investigation further by performing western blotting.

**Figure 2:**
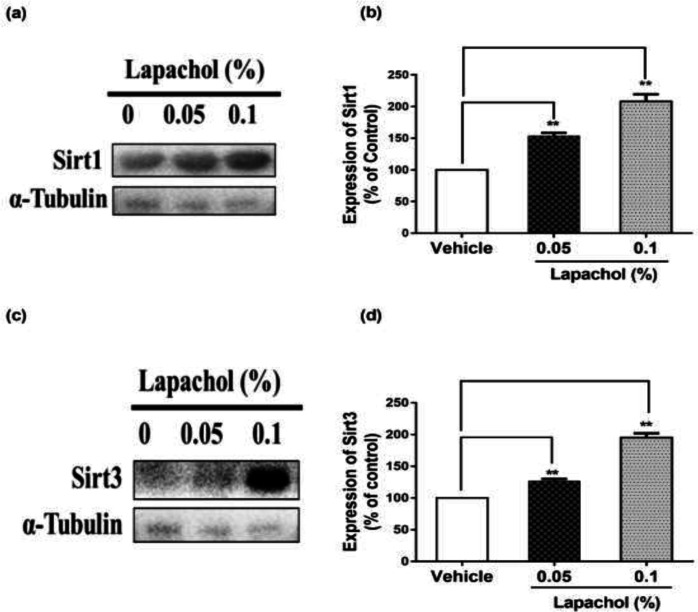
Lapachol enhances sirt1/sirt3 expression in mice skin sample. Skin samples from five mice per group were collected. Western blot was performed on the very next day of skin collection. (a) Western blot image showing expression of sirt1 in the skin of mice from lapachol and vehicle-treated groups. (b) Graphical representation of the expression pattern of sirt3. (c) Western blot image showing expression of sirt3 in the skin of mice from lapachol and vehicle-treated groups. (d) Graphical representation of the expression pattern of sirt3.Values are means ± SD^.^^∗∗^*p* < 0.01 and ^∗∗∗^*p* < 0.001 *vs.* vehicle/control group

The western blot results showed that sirt1 expression was increased significantly in mice treated with lapachol (Figure 2A). The diabetic wounded mice treated with solvent only showed low expression of sirt1 as compared to mice treated with lapachol 0.05% and lapachol 0.1%. There was a significant difference between sirt1 expressions in the vehicle as compared to the lapachol treated groups (Figure 2B). Mice treated with a 0.1% dose of lapachol showed the highest sirt1 expression followed by mice treated with 0.05% lapachol. Similarly, the sirt3 expression in lapachol treated groups was high than the vehicle-treated group (Figure 2C). Mice treated with lapachol 0.1% were found to have more sirt3 expression as compared to mice treated with lapachol 0.05%. Overall, both lapachol treated mice groups showed significantly higher expression of sirt3 as compared to the non-treated group (Figure 2D). This significantly increased sirt1 and sirt3 expression in lapachol treated mice groups back our hypothesis and provides an important clue for the role of these proteins in diabetic wound healing.

*Lapachol Increases the Expression of Migration and Angiogenesis-Related Proteins in Diabetic Mice Skin*

**Figure 3:**
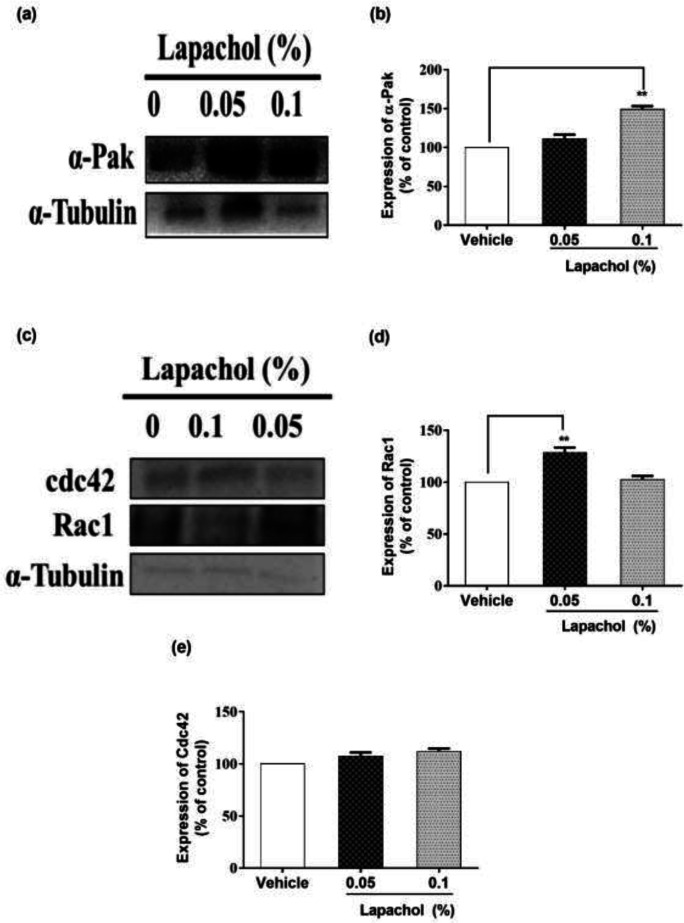
Effect of lapachol on Migration-related proteins. Five mice from each group were selected and sacrificed. Skin samples collected were subject to western blot analysis next day following the sacrifice. (a) Western blot image showing expression of α-pak in the skin of mice from lapachol and vehicle-treated groups. (b) Graphical representation of the expression pattern of α-PAK. (c) Western blot image showing expression of Rac1 and Cdc42 in the skin of mice from lapachol and vehicle-treated groups. (d) Graphical representation of expression pattern of RAC1 (e) Graphical representation of CDC42

We checked the expression of α-PAK protein by western blotting and it turned to be higher in mice treated with lapachol as compared to the mice treated with vehicle only. Mice treated with lapachol 0.1% showed significantly high expression of α-PAK (Figure 3A). However, the expression of α-PAK in mice treated with lapachol 0.05% was although higher as compared to the vehicle group, but the difference did not prove to be significant when analyzed statistically. (Figure 3B).

Similarly, we found that the expression of RAC1 protein was high in lapachol treated mice skin samples as compared to vehicle-treated mice skin samples. There was no significant difference between the expressions of RAC1 in mice treated with lapachol 0.1% and vehicle group (Figure 3C). However, the expression of RAC1 was significantly increased in mice treated with lapachol 0.05% as compared to vehicle (Figure 3D), confirming the role of lapachol in wound healing. Next, we ought to check the expression pattern of CDC42 in mice skin samples by western blotting. This time, we did not get any satisfactory results. The expression of this protein was although relatively high in lapachol treated group, specifically the lapachol 0.1% group, but the statistical analysis did not show any significant difference in comparison to the vehicle (Figure 3E). This part of the experiment needs further investigation and improvement. 

*Lapachol Enhances Skin Regeneration by Increased Growth Factor Release in Diabetic Mice*

**Figure 4:**
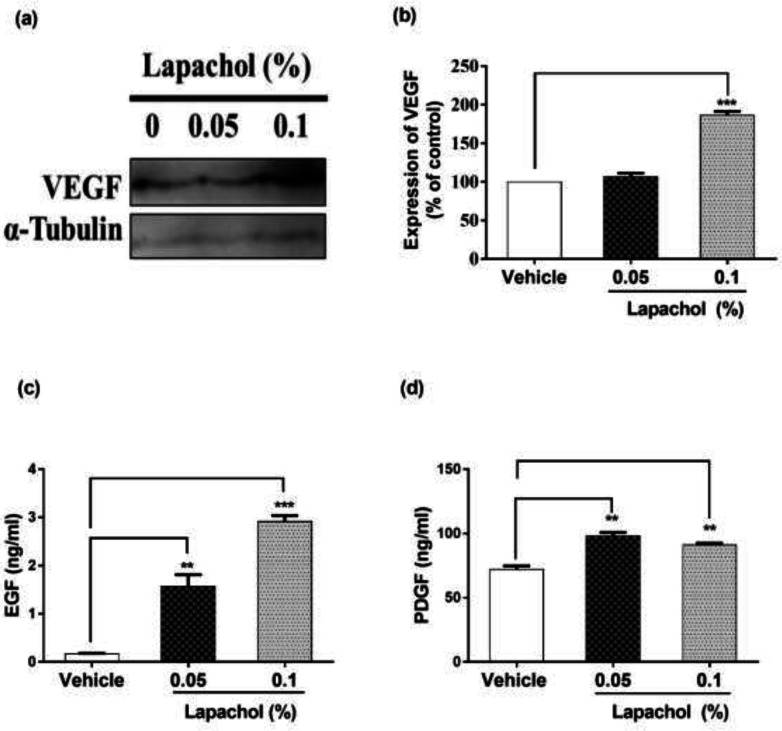
Effect of lapachol on angiogenesis and Growth factors. Five mice from each group were selected and sacrificed. Skin samples collected were subject to western blot analysis next day following the sacrifice for VEGF expression and ELISA for EGF and PDGF. (a) Western blot image showing expression of VEGF in the skin of mice from lapachol and vehicle-treated groups. (b) Graphical representation of the expression pattern of VEGF. (c and d) Mice treated with lapachol showed the highest concentration of EGF and PDGF as compared to vehicle. Values are means ± SD. ^∗∗^*p* < 0.01 and ^∗∗∗^*p* < 0.001 *vs. *vehicle/control group

The expression of VEGF was high in lapachol treated mice skin samples as compared to vehicle-treated mice skin samples (Figure 4A). There was a significant difference between expressions of VEGF protein in mice treated with lapachol 0.1% as compared to the mice treated with vehicle. However, no significant difference was observed between the expression pattern of VEGF in mice skin treated with 0.05% lapachol and mice treated with vehicle. But overall, lapachol treatment resulted in high expression of VEGF, supporting our claim of lapachol as an enhancer of migratory and angiogenic proteins, thus ameliorates diabetic wound healing (Figure 4B).

We performed ELISA for detecting the concentration of growth factors EGF and PDGF to further evaluate the wound healing potential of lapachol. Skin sample from five mice per group was collected for the experiment. Total cell protein was extracted from each mice group. There was a marked increase in the concentration of EGF in mice treated with lapachol as compared to the group treated with vehicle only. The concentration of EGF was significantly high in mice skin treated with lapachol 0.05% and lapachol 0.1% as compared to vehicle. Mice treated with lapachol 0.1% showed the highest concentration of EGF compared to vehicles. Mice skin obtained from the group treated with vehicle only gave very less concentration of EGF, almost negligible (Figure 4C). Similarly, lapachol treated mice showed a high concentration of PDGF as compared to the mice treated with vehicle only. There was a significant increase in the concentration of PDGF in lapachol treated groups (Figure 4D).

**Figure 5:**
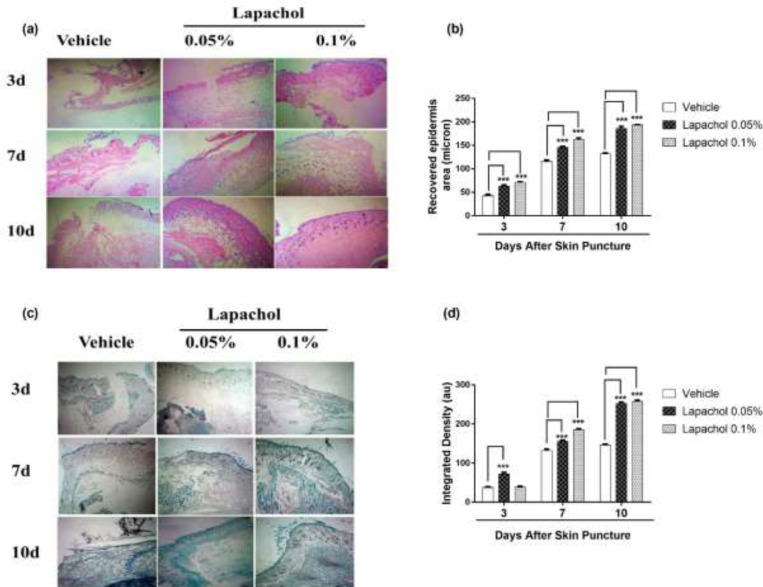
Effect of lapachol on epidermal formation and collagen deposition. Five mice from each group were selected and sacrificed. Skin samples collected were subject to Histological analysis next day. (a) H&E-stained skin tissue sections on day 3, 7 and day 10 -post-wounding to compare neo-epithelium generation. (b) Graphical representation of the recovered epidermal area in mouse skin on day 3, 7 and day 10 -post-injury. (c) Masson’s trichrome-stained sections show more collagen deposition in mouse skin treated with lapachol on day 3, 7 and 10 -post-wounding than vehicle-treated mice. (d) Graphical representation of collagen deposition in mouse skin on day 3, day 7 and day 10 -post-injury. Values are means ± SD.^∗∗^*p* < 0.01 and ^∗∗∗^*p* < 0.001 *vs. *vehicle/control group

Skin samples collected from 5 mice per group on days 3^rd^, 7^th^ and 10^th^ of the experiment were stained with Hematoxylin and Eosin (H&E) to check the effect of lapachol on skin regeneration, granulation tissue formation, and neuroepithelium formation. There was faster tissue regeneration and granulation tissue formation in mice treated with lapachol as compared to the vehicle-treated mice on day3, day7 and day 10. Both the dermal and epidermal layers were fully formed and well-characterized (Figure 5A). The vehicle group treated with solvent only didn’t show remarkable tissue regeneration, also there was no well-characterized formation of epidermal and dermal layers. Lapachol 0.1% treated mice showed a faster wound healing rate with a fully developed epidermal layer on day 7^th^ of treatment as compared to the 0.05% lapachol. However, both the lapachol 0.1% and 0.05% proved to have the same wound healing effect on day 10^th^. There was no significant difference between the two doses on day 10^th^, although they were significantly good in comparison to the vehicle on all three-time points (Figure 5B). The results obtained after H&E analysis strengthened our idea of the potential role of lapachol in diabetic wound healing, as skin regeneration can be observed (Figure 5A).

Further, we aimed to investigate the effect of different concentrations of lapachol on collagen deposition and organization. For this purpose, mice skin samples from all three groups were collected and stained with Masson’s trichrome stain. Intriguingly, the same results were obtained as stated previously. Mice treated with lapachol showed more collagen deposition shown in blue color with uniform and well-organized collagen as compared to the group of mice treated with vehicle only (Figure 5C). The amount of collagen was not only less in the vehicle-treated group but it was also haphazardly organized on both day 7 and day 10 post-injury. There was a significant difference between collagen deposition in mice skin treated with lapachol as compared to the vehicle-treated group on days 7^th^ and 10^th ^-post-injury. Mice treated with lapachol 0.1% showed more collagen deposition on day 7^th^ of treatment as compared to mice treated with lapachol 0.05%. However, the effect of both concentrations of lapachol on collagen content was almost the same on day 10^th^ of treatment (Figure 5D).

## Discussion

Diabetes mellitus is a metabolic disorder characterized by defect in insulin release, insulin working or may be both (34). For efficient wound healing, it is important for various types of cells to work in a coordinated fashion to ultimately restore the damaged skin (35). Chronic wound healing problems are often contributed by diabetes mellitus. The pathophysiological link between impaired wound healing and diabetes is very complicated and it depends on several factors (11). In the present study, we sought to perform a set of experiments to find a novel potential therapeutic agent that could enhance the wound healing process in a diabetic mouse model. A naphthoquinone lapachol was used to check its wound healing activity in diabetic mice. Lapachol has a wide spectrum of therapeutic properties (28). It is found to be anti-malarial, anti-tumor, and anti-oxidant along with other naphthoquinones such as lawsone and β-lapachone (36-39).

Two different concentrations of lapachol were used to treat diabetic wounded mice in comparison to mice treated with vehicle only. Lapachol showed promising wound healing effects *in-vivo*. The photographs taken digitally from day 0 to day 10^th^ showed satisfactory results. Wounds were almost completely closed on day 10 post-injury in mice skin treated with lapachol 0.1% and lapachol 0.5% as compared to vehicle (Figure 1). The data obtained from this experiment proved that lapachol is involved in wound repair. Next, we wanted to check the downstream target of lapachol. Previous studies state that increased epidermal sirt1 expression is associated with improved wound healing by regulating cell migration, re-epithelialization, inflammation, granulation, and redox response (22). It is already reported that another naphthoquinone derivative juglone is involved in sirt1 upregulation (22), that is why we opted for lapachol and checked for sirt1 and sirt3 as its potential targets. As expected, results obtained showed that lapachol treatment showed overexpression of sirt1 in mice skin treated with lapachol (Figures 2a and 2b). This was a remarkable finding. Next, we checked the role of lapachol in a probable expression of sirt3, which is an important protein for cell survival. Interestingly, we found a high expression of sirt3 in the skin of lapachol treated mice groups. It can be stated that lapachol is involved in increasing the expression of sirt3, as the sirt3 expression was very low in skin samples collected from mice treated with vehicle (Figures 2c and 2d).

Extending our experiments, we focused on different phases of the wound healing process and the cells involved in it. The skin epithelium recovery is initiated by cytoskeletal movements which are dynamic. RAC1 and CDC42 are small GTPases belonging to the Rho family and are known to be the key regulators of the cytoskeletal movements. They help in wound healing by accumulating in the wound edges (40, 41). Knowing the crucial role of these proteins, we checked their expression in mice skin samples by western blotting. Interestingly, our compound lapachol came out as a wound healing promoter in the diabetic mouse model by ameliorating the later phase processes of cutaneous wound healing like cell migration, angiogenesis, and tissue remodeling. This aim of ours is backed by the evidence we got by western blot analysis. Most probably, lapachol promoted these processes mainly by increasing the expression of proteins involved in cell migration including α-PAK/Rac1/Cdc42/ (Figures 3a-3e). This finding showed that lapachol enhanced wound healing by triggering skin cell migration as suggested in a recently published study that many of the novel derivatives of 1,4 naphthoquinone promotes wound closure by accelerating fibroblasts migration in diabetic conditions (31). The next step was to check the effect of lapachol on angiogenesis. Impaired blood vessel formation coupled with insufficient blood perfusion leads to complication in tissue repair in diabetics (42). We checked the effect of lapachol on VEGF. The decreased biological activity of VEGF in diabetic patients is due to extremely proteolytic surroundings of diabetic wounds (43). Lapachol treatment resulted in high expression of VEGF in skin taken from diabetic wounded mice. Mice skin taken from the group only treated with vehicle didn’t increase the VEGF concentration (Figures 4a and 4b). This increased expression of VEGF by lapachol treatment resulted in better wound healing as reported by Galiaon *et al*., that VEGF therapy ameliorates diabetic wound healing.

Growth factors are known to be involved in wound repair. The complex wound healing process is executed by a wide range of cytokines and chemokines (2). Thus, we checked the concentration of two important growth factors namely EGF and PDGF. Proteins extracted from skin samples collected on days 3^rd^, 7^th,^ and 10^th^ were checked for these growth factors by ELISA. We observed an increased concentration of EGF in both the groups that were treated with lapachol. The EGF concentration in vehicle-treated mice skin samples was very less, demonstrating the powerful effect of lapachol (Figure 4c). Similarly, PDGF concentration was also high in lapachol treated mice as compared to vehicle-treated mice. However, contrary to the previously obtained results, this time, the low concentration of lapachol was more effective than the high concentration (Figure 4d). This depicts that the concentration of lapachol is very critical while choosing the dose as in the case of juglone (44). Similar effects of lapachol were observed in tissue development rate as well. The epidermis and dermis were quickly restored in mice skin treated with lapachol as compared to the mice treated with vehicle only. From the results obtained, we thus demonstrate that lapachol can be used as a potent agent for dermal and epidermal regeneration (Figures 5a and 5b). 

To further assess the role of lapachol in wound healing, we stained the skin tissue with Masson’s trichrome stain and it was proved that lapachol is very helpful in ameliorating diabetic wound healing by increasing the amount of collagen content in the wound site. Lapachol treatment resulted in better collagen deposition in skin tissue collected on days 7^th^ and 10^th^ -post-injury. (Figures 5c and 5d).

Taken together these results, it is stated that though the experiments we performed for assessing the potential wound healing activity of lapachol gave spectacular results, still the study needs further validation and authentication. Most importantly, an optimum dose of lapachol must be found out for getting better results. Future studies are needed with more advanced techniques and methodologies.

## Conclusion

To conclude, our results suggest that lapachol is a novel potential sirt1 and sirt3 activating compound that ameliorates wound healing in the diabetic mouse model by promoting cell migration, cell proliferation, angiogenesis, and tissue remodeling. Lapachol mainly enhances wound healing by increasing the expression of Rac1/Cdc42/α-Pak proteins and thus can be regarded as a potential wound healing drug candidate and hence sirt1 and sirt3 as potential drug targets for diabetic wound healing. Our findings also give a clue about how critical the concentration of lapachol is because we got varying results at two different doses. Further studies are required to understand the mechanism behind sirt1 and sirt3 activation and increased expression by lapachol treatment.
